# Effect of *Trataka* (Yogic Visual Concentration) on the Performance in the Corsi-Block Tapping Task: A Repeated Measures Study

**DOI:** 10.3389/fpsyg.2021.773049

**Published:** 2021-12-17

**Authors:** P. S. Swathi, Raghavendra Bhat, Apar Avinash Saoji

**Affiliations:** Division of Yoga and Life Sciences, Swami Vivekananda Yoga Anusandhana Samsthana, Bengaluru, India

**Keywords:** *Trataka*, yoga, shatkriya, kriya, Corsi-block tapping task, eye exercise, cognition, spatial memory

## Abstract

**Background and Objective:** Attention and memory are essential aspects of cognitive health. Yogasanas, pranayama, and meditation have shown to improve cognitive functions. There has been no assessment of *Trataka* (yogic visual concentration) on working or on spatial memory. The present study was planned to assess the immediate effects of *Trataka* and of eye exercise sessions on the Corsi-block tapping task (CBTT).

**Methods:** A total of 41 healthy volunteers of both genders with age 23.21 ± 2.81 years were recruited. All participants underwent baseline assessment, followed by 2 weeks of training in Trataka (including eye exercise). Each training session lasted for 20 min/day for 6 days a week. After completion of the training period, a 1-week washout period was given. Each participant then was assessed in two sessions in *Trataka* and in eye exercise on two separate days, maintaining the same time of the day. Repeated measure analysis of variance with Holm’s adjustment was performed to check the difference between the sessions.

**Results:** Significant within-subjects effects were observed for forward Corsi span andforward total score (*p* < 0.001), and also for backward Corsi span (*p* < 0.05) and backward total score (*p* < 0.05). *Post hoc* analyses revealed *Trataka* session to be better than eye exercises and baseline. The eye exercise session did not show any significant changes in the CBTT.

**Conclusion:** The result suggests that *Trataka* session improves working memory, spatial memory, and spatial attention.

## Introduction

Yoga, an ancient Indian tradition, is aimed at all-round personality development (Taimni, 2010). The practices in the discipline of yoga include yama and niyama (moral and ethical conduct), asana (physical postures), pranayama (regulated breathing), dharana, dhyana (meditation), and shuddhikriya (cleansing practices). Scientific research in recent times has explored the positive impact of yoga practices on various domains of physiology and psychology in healthy and therapeutic settings (Field, 2016). One major area of interest in yoga research has been the effects of yoga practices on cognition and performance. Yoga practices appear to prevent neurodegeneration and enhance neuroplasticity by influencing specific brain areas involved with domains of cognition such as hippocampus, amygdala, prefrontal cortex, insula, and default mode network (Marciniak et al., 2014; Gothe et al., 2019). A meta-analysis, which included fifteen RCTs and eight acute exposure studies, indicated the beneficial effect of yoga on cognition, attention, processing speed, and memory (Gothe and McAuley, 2015).

Various aspects of cognition, such as spatial and visual memory scores (Joshi and Telles, 2008; Garg et al., 2016; Gupta et al., 2019), verbal memory (Naveen et al., 1997), executive functions, attention, and concentration (Chattha et al., 2008), working memory (Subramanya and Telles, 2009), response inhibition (Rajesh et al., 2014), visual attention (Jarraya et al., 2019), and task-switching (Anusuya et al., 2021), were found to be positively influenced through yoga practices such as yogasanas, pranayama, and meditation techniques. Yoga practice was found to be better than physical exercises in improving cognitive functions in school children (Vhavle et al., 2019).

The classical texts of *Hathayoga* described the profound impact of the six cleansing techniques on various aspects of one’s personality, which are also validated through empirical studies (Muktibodhananda, 1999; Swathi et al., 2020). *Trataka* (Yogic Visual concentration) is one of the cleansing techniques considered to enhance vision and positively influence cognitive processes. Since the process of *Trataka* involves focused attention on a candle flame, the practice leads to the mind becoming one-pointed and arouses inner vision (Muktibodhananda, 1999). Earlier studies on *Trataka* and cognition have demonstrated enhanced performance in Stroop Task (Raghavendra and Singh, 2016; Sherlee and David, 2020), Six Letter Cancelation, Trail Making tasks (Talwadkar et al., 2014), and Critical Flicker Fusion (Mallick and Kulkarni, 2010). Considering the earlier studies on *Trataka* and cognition, we hypothesize that *Trataka* may positively influence the domains of cognition, such as spatial and working memory. Corsi-block tapping task (CBTT) is a neuropsychological test that measures visuospatial short-term and working memory. The task can be performed using a computer to collect the data with precision (Kessels et al., 2000; Siddi et al., 2020). Considering the wide use and ease of administration of CBTT, the current study was designed to evaluate the effect of *Trataka* on the performance in the CBTT.

## Materials and Methods

### Participants

A total of 90 volunteers from a University in South India were briefed about the study protocol. Out of which, 60 consented to participate in the study. The inclusion criteria were normal vision (6/6) on Snellen’s chart and regular physical and psychological health as assessed by a physician who, otherwise, had no role in the study. We included participants with prior experience of yoga practices other than *Trataka*. We excluded volunteers who had any known eye disorders, including refractive errors, color blindness, glaucoma, cataract, any ophthalmological surgeries, or presence of cognitive or neurological disorders, respiratory or cardiac, and sensory abnormalities. We also excluded participants who had a history of smoking or alcoholism. Finally, 41 subjects (8 male and 33 female) with their mean (± SD) age 23.21 ± 2.81 years were recruited to the study. Out of the 41 subjects, 31 were pursuing their undergraduate education, 4 were graduates, and 6 had completed postgraduation. Their experience in yoga ranged between 1 and 7 years (mean ± SD = 3.98 ± 1.44).

### Sample Size Calculation

The sample size was calculated using G*power where alpha was 0.05 and power was 0.8. The effect size was found to be 0.50 (Gupta et al., 2019). The recommended sample size resulted in being 33 participants for each session. Considering dropouts to be at about 25% during the training, we decided to have 41 participants for each session.

### Ethical Consideration and Trial Registration

The Institutional Ethics committee approved the study of the university (Ref. No: RES/IEC-SVYASA/182-C/2021). Written informed consent was obtained from individual participants before their recruitment to the study. The study was registered with the Clinical Trial Registry of India (CTRI/2021/03/031872).

### Trial Design

We executed a within-subject repeated measures design. All participants underwent baseline assessment, followed by 2 weeks of training in *Trataka* (including eye exercise). Each training session lasted for 20 min/day for 6 days a week. This orientation was administered to avoid individual variations in the practice. After completion of the training period, a 1-week washout period was given. Each participant then was assessed in two sessions in *Trataka* and in eye exercise on two separate days, maintaining the same time of the day (between 4 pm and 6 pm). The order of allotment of *Trataka* and eye exercise sessions was block randomized using a web-based random number generator^1^. Half the participants practiced *Trataka* on day 1, eye exercise on day 2 and vice versa. The CBTT was recorded following both the trial conditions (*Trataka* and eye exercise).

### Intervention

#### Baseline

The participants were asked to give their baseline assessment without any intervention. On the day of baseline assessment, the participants were seated comfortably in a cross-legged position for 5 min prior to the commencement of the CBTT performance.

#### *Trataka* Session

Each *Trataka* session consisted of 20 min practice. Throughout the practice, the participants were seated comfortably on the floor in a cross-legged position. The practice consists of 2 distinct stages. Each *Trataka* session involved a preparatory stage of eye exercises for 10 min. These were performed with eyes open in a well-lit, soundproofed recording room in the laboratory. During this stage, the participants were asked to move the eyeballs in horizontal, vertical, diagonal, and circular directions. The second stage is the practice of gazing at the candle flame in a dark room, where the candle was placed at the participant’s eye level at a distance of 2 m. The participants were asked to fix their gaze on the candle flame for about 2 to 3 min without blinking their eyes. Then they were asked to visualize the candle flame between the eyebrows with closed eyes. This process was repeated for three rounds. Later, subjects were asked to defocus, and practice concluded in silence with a prayer. This stage lasted for a total duration of 10 min. A pre-recorded audio was used to maintain uniformity of the practice among participants.

#### Eye Exercise Session

The eye exercise session included eyeball movements in the horizontal, vertical, diagonal, and circular directions for 10 min followed by 10 min of quiet sitting with closed eyes. The eyes open part was performed in well-lit room, while the eyes closed part was performed by switching off the lights to maintain similarity of interventions.

### Assessments

#### Corsi-Block Tapping Task

**FIGURE 1:**
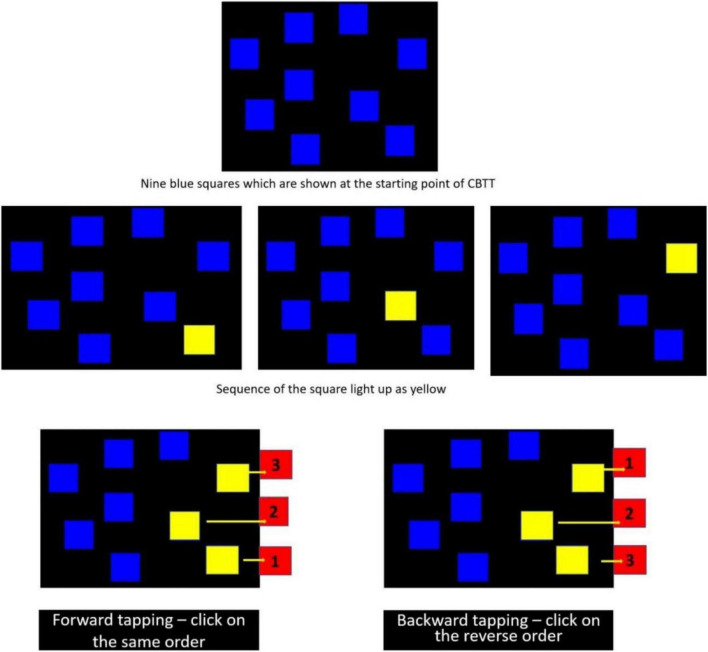
Corsi-block tapping task (CBTT): Forward and backward.

Corsi-block tapping task is a popular neuro-psychological task used to assess working and spatial memory. Nine blue squares appear on the screen. For each trial, the squares “light up” as yellow one by one in a varying sequence. After the presentation, the participants had to click each of the boxes in a similar order in which they have to “lit up” the first part of the task, i.e., forward tapping. In the second part of the task, they maintained the reverse order, i.e., backward tapping (Kessels et al., 2008). The task begins with a two-box sequence to a maximum of nine. The test gets terminated when the participant cannot remember the sequence for two consecutive trials at any one level. Hence, the test assesses the following four variables: (i) forward Corsi span, (ii) forward total score, (iii) backward Corsi span, and (iv) the backward total score. Figure 1 illustrates the forward and backward CBTT.

#### Presentation of Corsi-Block Tapping Task

We assessed the participants at baseline, following *Trataka* and eye exercise sessions. The participants were asked to avoid caffeine consumption on all the assessment days as it may alter their cognitive abilities. The CBTT (Kessels et al., 2000, 2008) was presented using the INQUISIT software package 4.0 (Millisecond Software, LLC, Seattle, WA, United States) on a Dell desktop computer with a 21.5 color monitor. Uniform configuration was maintained for the computers on which the CBTT was presented to maintain the uniform processing speed. All participants received a practice session prior to the actual assessment session to familiarize themselves with the CBTT. The experiment was conducted individually in a room under standard fluorescent lighting in the research laboratory.

#### Data Analysis

The data were tabulated and data analyses were performed using JASP statistical package version 0.14.1^2^. The data were tested for normality and repeated measures (RM) ANOVA for within-subjects effects. *Post hoc* corrections were done using Holm’s method for checking the differences between sessions.

## Results

All 41 participants (eight male) completed all three sessions. RM ANOVA demonstrated significant within-subjects effect in Forward Corsi Span *F*(2,80) = 8.757, *p* < 0.001; Forward total scores *F*(2,80) = 11.377, *p* < 0.001; Backward Corsi Span *F*(2,80) = 3.629, *p* = 0.031; Backward total scores *F*(2,80) = 3.950, *p* = 0.023. The within-subjects effects obtained through RM ANOVA are presented in Table 1.

**TABLE 1:** Results of repeated measures analysis of variance for within-subjects effects.

Variable	*F*	df	*P*	Partial η^2^
Forward Corsi span	8.757	2, 80	<0.001	0.180
Forward total score	11.377	2, 80	<0.001	0.221
Backward Corsi span	3.629	2, 80	=0.031	0.083
Backward total score	3.950	2, 80	=0.023	0.090

Pairwise comparisons between the sessions performed through RM ANOVA with Holm’s corrections demonstrated significantly higher scores following *Trataka* sessions when compared with baseline for Forward Corsi Span, *t* = −4.11, *p* < 0.001; Forward Total Score, *t* = −4.76, *p* < 0.001; and Backward Total Score, *t* = −2.74, *p* < 0.05. Scores following the *Trataka* session were significantly higher than following Eye exercises for Forward Corsi Span, *t* = 2.74, *p* < 0.05; Forward Total Score, *t* = 2.65, *p* < 0.05. The scores increased from baseline, following Eye exercise only for Forward Total Scores, *t* = −2.10, *p* < 0.05. The effect sizes and *t*-values for between sessions using RM ANOVA with Holm’s correction along with the group mean and SD are reported in Table 2.

**TABLE 2:** Pairwise comparisons between sessions for the performance in Corsi-block tapping task (CBTT) using repeated measures ANOVA with Holm’s Corrections.

Variables	Baseline	*Trataka*	Eye exercise	Baseline vs. *Trataka*			Baseline vs. Eye exercise			Trataka vs. Eye exercise		
				*t* value	*p* value	Cohen’s *d*	*t* value	*p* value	Cohen’s *d*	*t* value	*p* value	Cohen’s *d*
Forward Corsi span	5.5 ± 0.8	6.1 ± 0.9	5.7 ± 1.0	−4.11	<0.001	0.642	−1.37	=0.17	0.214	2.74	<0.05	0.428
Forward total score	44.2615.59	56.95 ± 17.77	49.87 ± 18.60	−4.76	<0.001	0.743	−2.10	<0.05	0.329	2.65	<0.05	0.415
Backward Corsi span	5.9 ± 0.4	6.1 ± 0.4	5.8 ± 0.7	−2.22	=0.06	0.348	0.20	=0.84	0.032	2.43	=0.052	0.379
Backward total score	51.41 ± 10.67	56.68 ± 10.91	52.97 ± 11.67	−2.74	<0.05	0.427	−0.81	=0.42	0.127	1.92	=0.115	0.301

## Discussion

The current study was designed to elicit if the practice of *Trataka* affects the working and the spatial memory through the performances in the CBTT. All the four measures, viz., forward and backward Corsi spans, and total scores, demonstrated significance within the subject’s effect. The Corsi span and total scores were higher following *Trataka* while comparing with baseline and Eye exercises. Scores following eye exercises and baseline sessions were insignificant except in Forward total score. The forward span and total score of CBTT measure material-specific slave systems. The backward test measures primarily tax central executive resources (Monaco et al., 2013). Thus, improvements in both forward and backward span and total scores indicated a positive effect of *Trataka* on working, spatial memory, and executive functions while comparing with baseline and eye exercises.

Earlier studies on *Trataka* and cognition have shown improvements in the domains of selective attention, cognitive flexibility, and response inhibition through the Stroop task (Raghavendra and Singh, 2016; Sherlee and David, 2020). Another study shown improvement in the performance of critical flicker fusion after the immediate practice of *Trataka* in 35 volunteers (Mallick and Kulkarni, 2010). After the practice of *Trataka* for 26 days, the performances of the digit span test, the six-letter cancelation test, and the trail making test significantly improved in thirty elderly subjects compared to the waitlist control group (Talwadkar et al., 2014). Thus, improvements noted in our study in the cognitive abilities following *Trataka* are similar to the earlier studies.

*Trataka* practice is indicated to positively influence cognition from both classical texts of yoga (Muktibodhananda, 1999) and empirical studies (Swathi et al., 2020). Although classified as a cleansing procedure, the practice of *Trataka* is similar to focused meditation techniques. Earlier studies on meditation for a total duration of 8 weeks shown decreased negative mood and enhanced attention, working memory, and decreased state anxiety on the trier social stress test (TSST) in population naive to meditation practice (Basso et al., 2019). Another focused attention meditation showed significant improvement in working memory in the reading span test and in activation of bilateral dorsolateral prefrontal cortex (DLPFC) during the intervention in the experimental group (Yamaya et al., 2021). Similarly, other Yoga interventions showed improved cognitive communicative abilities (Namratha et al., 2017).

The improvement in the performance of CBTT may have been mediated through relaxation, attained through the practice of *Trataka* (Raghavendra and Ramamurthy, 2014). The possible mechanisms for improving working and spatial memory following the *Trataka* session could be the process of *Trataka* itself, involving focused attention. This focused attention is also elaborated in the *Yoga Sutras* (aphorisms) of *Patanjali* (Taimni, 2010). A recent study has also demonstrated enhanced mindfulness, attention, and reduced mind-wandering with the practice of *Trataka* (Swathi et al., 2021). Thus, improved working memory found in our study could be due to a reduction in mind-wandering and enhanced focusing. The prefrontal cortex is associated with memory, attention, executive functions, and various other complex cognitive functions (Miller, 2000). Thus, the results following the *Trataka* session could be due to activation of the prefrontal cortex. However, further studies with neuroimaging techniques may be required to confirm this mechanism of action.

Another possible mechanism could be a surge in melatonin release due to practice in the dim light. It is known that bright light tunes the suprachiasmatic nucleus (SCN) that regulates the circadian rhythm. Exposure to bright light impedes the melatonin synthesis, whereas the dim light initiates the surge in melatonin release (Zisapel, 2018). Melatonin has been found to positively influence learning and memory (Zakaria et al., 2016). Thus, further studies on *Trataka* may assess the serum melatonin levels as one of the variables.

Our study indicated a beneficial role of *Trataka* in enhancing the CBTT performance in healthy volunteers. CBTT performance is commonly altered in neurodegenerative disorders such as early-stage Parkinson’s (Stoffers et al., 2003) and Alzheimer’s disease (Guariglia, 2007). Thus, future studies may be planned in a clinical population, where the CBTT performance is compromised.

Using a repeated measures design for immediate effect is one of the strengths of the study. We also used a computer-based program to execute CBTT, which enabled robust results (Brunetti et al., 2014). The limitation of the study includes not incorporating a neuro-imaging technique, which has limited our ability to predict the exact mechanism of action. Thus, future studies on *Trataka* and cognitive performance should include neuroimaging techniques. Another major limitation of the study is control condition which had eye exercise for 10 min followed by 10 min of quiet sitting in which they were told not to meditate. However, we are not sure if during quiet sitting, if they focused on breathing or let their mind wandered freely. We could not get an equal number of male and female participants and were also unable to study the impact of long-term practice of *tataka*. Lastly, the effect of *Trataka* in a population with mild cognitive decline could be studied in future.

## Conclusion

The results of this study indicated a positive impact of the *Trataka* session on the CBTT, indicating enhanced working memory, spatial memory, and spatial attention among the subjects compared to the baseline and eye exercise sessions. Thus, *Trataka* could be used to improve memory and attention in young adults.

## Data Availability Statement

The raw data supporting the conclusions of this article will be made available by the authors, without undue reservation.

## Ethics Statement

The studies involving human participants were reviewed and approved by Swami Vivekananda Yoga Anusandhana Samsthana. The patients/participants provided their written informed consent to participate in this study.

## Author Contributions

PS was involved in conceptualization, execution, data collection, and writing and editing of the manuscript. RB was involved in conceptualization, data analysis and interpretation, and writing and editing of the manuscript. AS was involved in conceptualization, data collection, analysis and interpretation, and writing and editing of the manuscript. All authors contributed to the article and approved the submitted version.

## Conflict of Interest

The authors declare that the research was conducted in the absence of any commercial or financial relationships that could be construed as a potential conflict of interest.

## Publisher’s Note

All claims expressed in this article are solely those of the authors and do not necessarily represent those of their affiliated organizations, or those of the publisher, the editors and the reviewers. Any product that may be evaluated in this article, or claim that may be made by its manufacturer, is not guaranteed or endorsed by the publisher.

## Footnotes

1
www.randomizer.org

2
https://jasp-stats.org
